# Molecular Mechanisms Underlying Defense Responses of Potato (*Solanum tuberosum* L.) to Environmental Stress and CRISPR/Cas-Mediated Engineering of Stress Tolerance

**DOI:** 10.3390/plants14131983

**Published:** 2025-06-28

**Authors:** Maxim Sutula, Dilnur Tussipkan, Balnur Kali, Shuga Manabayeva

**Affiliations:** 1 Plant Genetic Engineering Laboratory, National Center for Biotechnology, Astana 010000, Kazakhstan; m.sutula@biocenter.kz (M.S.); tussipkan@biocenter.kz (D.T.); kali@biocenter.kz (B.K.); 2 Faculty of Natural Sciences, L.N. Gumilyov Eurasian National University, Astana 010000, Kazakhstan

**Keywords:** *Solanum tuberosum*, CRISPR/Cas, defense mechanisms, environmental stresses, secondary metabolites

## Abstract

Environmental stresses, such as drought, salinity, and pathogen attacks, significantly affect potato growth, development, and yield by disrupting key physiological and biochemical processes. Plant responses to these stresses are mediated by changes in gene expression, transcriptional regulation, and the activity of various functional proteins, all of which contribute to the molecular mechanisms of stress tolerance. Genome editing using the CRISPR/Cas9 system has been effectively used to enhance the resistance of potato to environmental stresses and to improve its nutritional value. This article provides a comprehensive review of recent studies retrieved from academic databases focusing on the effects of various environmental stressors on potato growth, yield, and postharvest storage. It also examines the influence of these stresses on the production of secondary metabolites and their associated molecular pathways. Finally, the review highlights advancements in the application of CRISPR/Cas-based genome editing technologies between 2021 and 2025 to improve stress tolerance and nutritional traits in potato plants.

## 1. Introduction

The potato (*Solanum tuberosum* L.) is the fourth most important food crop globally in terms of human consumption, following maize, wheat, and rice. It is cultivated in over 150 countries [1] and more than 50% of the total potato crop is grown in countries prone to drought and heat. Global production exceeds 370 million tons per year, with major producers including China, India, and Russia [2]. Due to abiotic stress, total potato yield loss is expected to reach up to 32% by 2050 [3]. The potato’s high yield, short growing cycle, and adaptability are essential for both subsistence and commercial agriculture. Nutritionally, potatoes are rich in carbohydrates, fiber, vitamin C, potassium, and antioxidants [4]. They provide a low-fat, cholesterol-free dietary staple. As a crop, potatoes support all four pillars of food security: availability, accessibility, utilization, and stability. However, their productivity is highly susceptible to environmental stresses, which significantly affect crop performance, tuber quality, and overall food security [5]. These stresses are classified as either biotic or abiotic, both of which contribute to substantial yield losses. Abiotic stresses, such as drought, heat, salinity, cold, and nutrient imbalances, are particularly detrimental to potatoes. These stresses often trigger genotoxic and oxidative damage, impairing DNA integrity and other cellular functions. The shallow root system of the potato exacerbates its sensitivity to water deficits, resulting in reduced photosynthesis, impaired tuberization, and early leaf senescence [6]. Heat stress during tuberization negatively affects starch accumulation and causes defects, such as knobbiness and hollow hearts [7], while salinity stress disrupts ion balance and water uptake, stunting growth and diminishing yields [8]. Similarly, cold and freezing stress damage cell membranes, reduce photosynthetic efficiency, and delay development [9]. Biotic stresses, including viral, fungal, and insect attacks, further compound these challenges. Late blight, caused by *Phytophthora infestans*, is the most devastating disease and can destroy entire crops under favorable conditions [10]. Insect pests, such as the Colorado potato beetle and aphids, inflict direct tissue damage and serve as vectors for viruses, such as potato virus Y (PVY) and potato leafroll virus (PLRV), thereby increasing disease pressure [11,12]. To survive under diverse and complex stress conditions, potato plants activate an array of defense mechanisms involving reprogramming gene expression and protein function. Transcription factors (TFs) play a central role in regulating plant defense responses to environmental stresses. They act as molecular mediators that sense external signals and activate the expression of defense-related genes downstream. They do so through intricate cross-talk with multiple signaling pathways, including those mediated by abscisic acid (ABA), salicylic acid (SA), jasmonic acid (JA), and reactive oxygen species (ROS). These hormones play a crucial role in plant adaptation by regulating the expression of stress-responsive genes, enhancing antioxidant defense mechanisms, and stimulating the biosynthesis of protective secondary metabolites [13,14]. TFs coordinate a wide range of physiological responses, such as the accumulation of protective metabolites and the induction of stress-responsive gene networks. The dynamic interplay between different TF families, such as bHLH, MYB, and WRKY, is essential for fine-tuning plant adaptation to both biotic and abiotic stresses. In 2021, Rahil Shahzad et al. [15] provided a comprehensive overview of the functional diversity and importance of transcription factor (TF) families in improving crop resilience through genetic and biotechnological interventions. This is especially relevant for potatoes, as they have limited genetic diversity for stress tolerance. Consequently, advanced breeding strategies, such as genome-wide association studies (GWASs), marker-assisted selection, and CRISPR/Cas9-mediated genome editing are increasingly being used more frequently to develop stress-resilient potato varieties that can withstand future climate uncertainties [16,17].

CRISPR/Cas9 technology is based on the use of the natural adaptive immunity of prokaryotes, which evolved over time. Originally discovered by Japanese scientists [18], these unusual repeats of genetic elements became an effective tool for DNA editing once the mechanism of action of the Cas9 protein was elucidated by the group of Doudna and Charpentier’s group [19]. The engineered CRISPR/Cas system enables the precise modification of target loci within complex plant genomes. CRISPR-based technologies have been successfully applied to genome engineering in both model plants and crop species, supporting a wide range of basic and applied research efforts [20]. Identifying genes associated with stress tolerance in potatoes and elucidating their regulatory mechanisms is critical for breeding new potato germplasm. Susceptibility genes (S-genes) play a critical role in mediating plant responses to stress. Their targeted modification using CRISPR/Cas technology offers a promising strategy to enhance stress tolerance in crops, including potatoes. Table 1 presents the most recent data on the application of CRISPR/Cas-based genome editing technologies from 2021 to 2025 to enhance stress tolerance and improve nutritional traits in potato plants. The CRISPR-associated Cas9, Cas12, and Cas14 act as endonucleases and can cleave double-stranded DNA or single-stranded (ss) DNA according to the guide RNA. This allows for the precise editing of specific regions of the genome [21]. RNA-guided RNA-targeting endonucleases, such as Cas13, can uniquely cleave the single-stranded (ss) RNA, including viral genomes [22,23,24]. Recent advances in CRISPR-based approaches have also facilitated the development of transgene-free genome-edited (GE) plants [25,26,27].

Understanding the physiological and molecular responses of potato to stress is critical to mitigating climate-induced yield losses and ensuring food security in a changing environment. Many researchers are actively studying this issue and developing and testing various strategies to improve crop resilience to environmental stress. In this review, we first discuss how various environmental factors, such as drought, salinity, and fungal and viral infections, impact potato growth, productivity, and postharvest quality. Next, we explore how these stressors influence the production of secondary metabolites, and the molecular mechanisms involved. Lastly, we discuss the potential of CRISPR/Cas-mediated genome editing to increase stress tolerance and improve the nutritional value of potato plants.

## 2. The Impact of Abiotic Stresses on Potato and the Application of CRISPR/Cas System to Enhance Tolerance

Abiotic stresses pose a significant challenge to the sustainable production of potatoes, greatly affecting yield and quality. Recent advances in genome editing technologies, particularly the CRISPR/Cas system, offer promising solutions for improving stress tolerance in potatoes through precise genetic modifications. Drought stress is an important abiotic factor that significantly impacts plant growth and productivity. *S. tuberosum*, a globally important food crop, is considered sensitive to drought due to its shallow root system and high water requirements, particularly during tuber development. As climate change increases the frequency and severity of drought events, improving drought tolerance in potatoes has become critical to maintaining crop yields and ensuring global food security. Recent studies have shown that drought stress in potatoes affects morphological, physiological, biochemical, and molecular processes, leading to poor plant performance and reduced tuber yield [30,59,60,61]. Morphological characteristics of potatoes, including plant height, root length and architecture, and tuber size and mass, significantly decrease under stressful conditions. Significant research advances have been achieved regarding the root system architecture of potatoes under different stress conditions, as reviewed by Zinta et al. [62]. It has been reported that the combination of drought stress and high temperatures has the greatest impact on morphological plant parameters, such as plant height, stem dry mass, leaf area, and root dry mass [63]. The physiological impact of abiotic stress on potatoes includes stomatal conductance, photosynthesis, and osmotic regulation. Drought-induced stomatal closure conserves water by reducing transpiration. However, this also restricts the diffusion of CO_2_ into the leaf, which can limit the substrate available for the Calvin cycle. Biochemically, heat and drought stress cause nutritional imbalances, leading to the excessive accumulation of reactive oxygen species (ROS) and superoxide anion radicals in plants. This results in oxidative damage to proteins, nucleic acids, and cell membranes. Furthermore, tolerant genotypes typically exhibit improved water use efficiency and better root architecture. They also accumulate oxidant enzymes, such as superoxide dismutase (SOD), peroxidase (POD), catalase (CAT), and thioredoxin peroxidase (TPX). These enzymes play important roles in the antioxidant defense system of plants [2,60,64]. The response of potato plants to the physiological and biochemical effects of heat, drought, and combined stress during the seedling stage was discussed in detail by Wang et al. in 2024 [30]. At the molecular level, potato plants utilize a complex regulatory network involving transcription factors, hormone signaling pathways, and metabolic enzymes. Key genes, including *StbHLH47*, *StLike3*, and *StDMR6-1*, modulate ABA signaling, auxin biosynthesis, and ROS detoxification, as well as stress-responsive transcription factors, such as DREB, NAC, and WRKY.

Basic helix-loop-helix (bHLH) TFs are a large family of regulatory proteins that play critical roles in plant development, growth, and responses to abiotic stress [65]. Specifically, bHLH TFs negatively regulate drought tolerance in potato, as reported by Wang et al. [66]. These findings highlight *StbHLH47* as a promising target for genome editing to improve drought tolerance in potato cultivars. Bioinformatics analysis suggests that the protein predominantly consists of α-helices and is localized to the nucleus, which is consistent with its role in transcriptional regulation. Promoter analysis has revealed several cis-acting elements associated with hormone and stress responses, including ABA-responsive elements (ABREs), MYB elements, MYC elements, and SA-response elements (TCA elements) [67,68,69].

**Figure 1:**
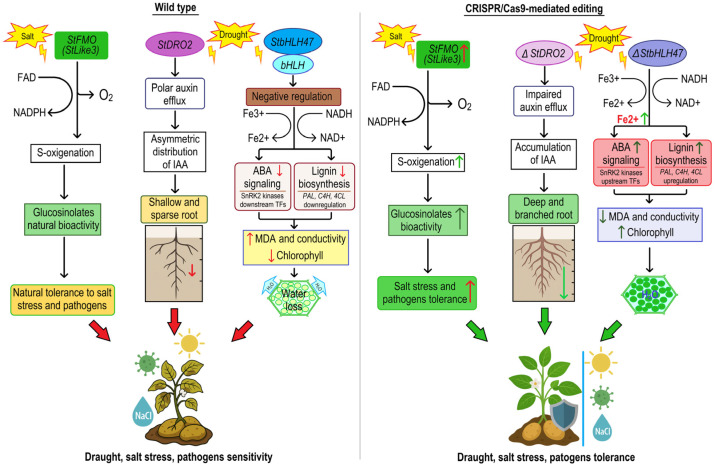
Molecular mechanism of *StbHLH47*, *StLike3*, *StDRO2*, and its role in drought and salt stress tolerance. In wild-type potato (**left panel**), *StLike3* is proposed to contribute to sulfur metabolism and oxidative stress responses. *StDRO2*, a membrane-localized auxin transporter of the DRO1 family, facilitates polar auxin efflux in root tissues, maintaining optimal auxin gradients for gravitropic responses and lateral root emergence. Functional *StDRO2* ensures balanced root architecture with moderate depth and branching. *StbHLH47* acts as a negative regulator of drought tolerance by suppressing ABA signaling and lignin biosynthesis, leading to weaker cell wall reinforcement, impaired antioxidant defense, and increased susceptibility to membrane damage and chlorophyll loss. In addition, *StbHLH47* represses iron uptake genes, resulting in suboptimal Fe(II) accumulation, which limits antioxidant capacity and exacerbates oxidative stress under drought conditions. In CRISPR/Cas9-edited plants (**right panel**), the overexpression of *StLike3* can enhance defense metabolism, while knockout of *StbHLH47* derepresses ABA-responsive and lignin biosynthetic pathways, improving cell wall integrity, antioxidant activity, and drought tolerance. Deregulation of iron homeostasis also leads to increased Fe(II) content, which supports chlorophyll stability, efficient photosynthesis, and enhanced ROS scavenging, thereby enhancing overall stress tolerance. Knockout of *StDRO2* reduces auxin efflux, leading to intracellular IAA accumulation in root apices. This stimulates lateral root initiation and elongation via ARF-mediated transcriptional cascades, producing a deeper and more branched root system. These architectural changes significantly improve water uptake under drought and confer enhanced tolerance without compromising growth.

However, Chauhan et al. [29] successfully generated *StbHLH47* knockout lines in *S. tuberosum* using CRISPR/Cas9 technology and observed altered expression genes involved in iron homeostasis. Specifically, the mutant lines exhibited reduced ferric chelate reductase (FCR) activity, yet they showed an increased expression of genes related to iron uptake, such as *StNAS4*, *StOPT3*, and *StFRO3*, leading to significantly higher accumulation of Fe(II) in tuber tissues. Although these results did not directly assess drought tolerance, the observed changes in iron homeostasis suggest a potential link to stress physiology. Furthermore, iron homeostasis is closely linked to ABA signaling. Under osmotic stress, ABA levels increase and facilitate iron redistribution through the regulation of iron transporter genes, thereby enhancing stress resistance. In chloroplasts, iron is essential for maintaining chlorophyll content and photosynthetic efficiency, both of which are critical under drought and salt stress [70,71]. In addition, other studies have demonstrated improved tolerance to various stressors by using CRISPR/Cas9 to knock out *bHLH* TFs in various species (Figure 1) [72,73].

Many recent reports have implicated flavin-containing monooxygenases (FMOs) in auxin biosynthesis, glucosinolate metabolism, and responses to abiotic and biotic stresses [74,75]. For example, specific FMOs in *Arabidopsis*, such as *FMOGS-OX1* to *FMOGS-OX7*, catalyze the S-oxygenation of methylthioalkyl glucosinolates to form methylsulfinylalkyl glucosinolates. This modifies the bioactivity of glucosinolates and contributes to plant defense against pathogens and pests [76]. In *S. tuberosum* (potato), the gene *StFMO* GS-OX-Like3 (*StLike3*) encodes a protein homologous to these FMOs. Although the specific biochemical role of *StLike3* in potato metabolism has not been fully elucidated, it is thought to be involved in the modification of sulfur-containing compounds, potentially affecting plant defense mechanisms and stress responses. The study conducted by Ye et al. [28] focused on improving genome editing efficiency in the potato cultivar CIP 149 by applying salt and osmotic stress during the CRISPR/Cas9-mediated editing of the *StLike3* gene. The results demonstrated that the mutation efficiency increased significantly when NaCl concentrations exceeded 20 mM and mannitol concentrations exceeded 100 mM, with the overall mutation efficiency exceeding 75%. The highest efficiency (91.67%) was observed at 50 mM NaCl, with no off-target mutations detected. Various types of mutations were identified, including chimeric mutations ranging from 62.50% to 100%, deletions up to 213 bp, bi-allelic mutations at 21.43% under mannitol and 35.5% at 10 mM NaCl, and single base insertions and replacements. Although no off-target effects were observed, root regeneration was partially inhibited at higher concentrations of NaCl and mannitol (Figure 1) [28].

The *StDRO2* gene in *S. tuberosum* encodes an auxin transporter that belongs to the DRO1 (Deeper Rooting 1) family. This transporter modulates polar auxin transport, thereby regulating root architecture [77,78,79]. *StDRO2* localizes to root cell membranes, directing auxin efflux from the pericycle and vascular tissues. This process controls root gravitropism and lateral root formation by ensuring the asymmetric distribution of indole-3-acetic acid (IAA) and enabling roots to adapt to environmental cues, such as soil moisture. Zhao et al. (2025) [77] reported that a natural splicing defect in intron 1 of *StDRO2* produces a non-functional variant that reduces auxin efflux, leading to IAA accumulation in root meristems. Consequently, these plants exhibited deeper, more branched root systems and improved drought tolerance compared to wild-type controls. This natural splicing variation establishes *StDRO2* as a valuable target for breeding drought-resilient potatoes.

## 3. Impact of Biotic Stresses on Potato and the Application of CRISPR/Cas System to Enhance Tolerance

Biotic stresses, such as infections caused by viruses, bacteria, fungi, and insect pests, pose a significant threat to potato cultivation worldwide. These stresses reduce yield and compromise tuber quality and marketability. The CRISPR/Cas genome editing system is a powerful tool for developing disease-resistant potato varieties because it enables the precise modifications of genes associated with immune responses and pathogen susceptibility.

### 3.1. Late Blight Disease

**Figure 2:**
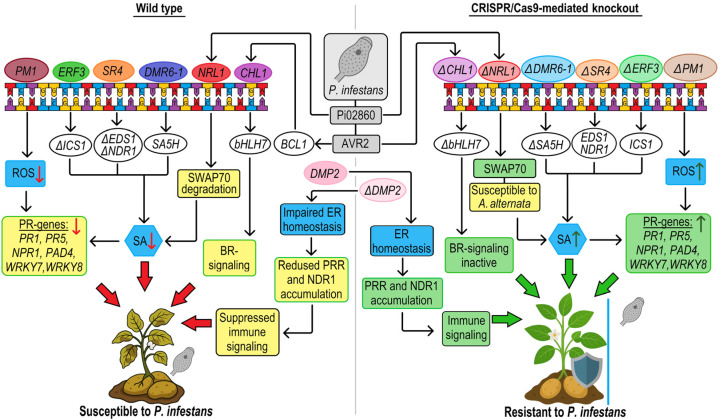
Molecular mechanism of *ERF3*, *SR4*, *DRM6-1*, *CHL1*, *NRL1*, *PM1*, *DMP2* and their role in resistance to *P. infestans.* Wild type potato (**left panel**), which negatively regulates the expression of defense-related genes: *ERF3*—suppresses defense gene activation downstream of ethylene signaling (*ISC1*); *SR4*—represses SA-mediated pathways by inhibiting *EDS1* and *NDR1*; *DMR6-1*—hydroxylates SA (*SA5H*), attenuating SA accumulation; *CHL1*—BR-responsive transcription factor that downregulates immunity under pathogen effector-triggered brassinosteroid signaling; *NRL1*—mediates proteasomal degradation of the immune regulator SWAP70; and *PM1*—suppresses PRR signaling at the plasma membrane and negatively modulates the expression of defense-related genes, including *StPR1*, *StPR5*, *StWRKY7*, and *StWRKY8*. These mechanisms weaken the immune response and facilitate pathogen colonization. *StDMP2* encodes an ER-localized DUF679 membrane protein that maintains endoplasmic reticulum (ER) homeostasis and facilitates proper folding and stabilization of immune receptors, such as PRRs and *NDR1*, thereby enhancing pattern-triggered immunity and hypersensitive response upon *Phytophthora* recognition. CRISPR/Cas9 knockouts (**right panel**) of these genes relieve transcriptional repression or eliminate enzymatic activities, resulting in enhanced SA signaling, increased expression of WRKY and *pathogenesis-related* genes (*PR1*, *PR5*), stabilization of immune regulators, and improved recognition of pathogen-associated molecular patterns. By contrast, CRISPR/Cas9-mediated loss-of-function of *DMP2* compromises ERQC, reduces PRR and *NDR1* accumulation, attenuates immune signaling, and increases susceptibility to *P. infestans*.

Late blight, caused by the oomycete pathogen *P. infestans*, is one of the most devastating diseases affecting potato production worldwide. Current disease management relies primarily on chemical fungicides, which pose significant environmental risks and raise concerns about sustainability and food security. Consequently, the development of genetically resistant potato varieties has become a critical strategy. Several resistance (R) genes, including *R3a*, *RGA2*, *RGA3*, *R1B-16*, *Rpi-blb2*, *Rpi*, and *Rpi-vnt1*, have been identified as conferring resistance to *P. infestans* [80]. Susceptibility (S) genes function as negative regulators of plant immunity, facilitating pathogenesis by suppressing host resistance mechanisms during biotic stress. Notable examples include *StNRL1*, *StERF3*, *StSR4*, and *StDMR6-1*, which are involved in complex hormonal and transcriptional networks that modulate defense responses in potatoes, primarily through SA and ethylene-dependent signaling pathways. Targeted genome editing of these regulatory genes offers a transformative opportunity; it not only enhances resistance to *P. infestans*, but also fundamentally re-engineers plant immune responses, providing more durable and broad-spectrum protection. Here, we analyze the biochemical mechanisms of key defense regulators and evaluate the phenotypic consequences of their targeted mutagenesis (Figure 2).

Turnbull et al. [81] described an important mechanism by which the *P. infestans* effector protein AVR2 interacts with the potato protein BSL1, a putative phosphatase involved in brassinosteroid (BR) signaling. This interaction leads to the up-regulation of BR-responsive genes, in particular, *StCHL1*, which encodes a bHLH transcription factor. The increased expression of *StCHL1* is associated with the suppression of plant immune responses, thereby increasing susceptibility to late blight. Knockdown of *StCHL1* transcripts using virus-induced gene silencing (VIGS) showed that loss-of-function mutations in *StCHL1* confer increased resistance to *P. infestans* without affecting plant development. This identifies *StCHL1* as a promising target for gene editing applications. In 2021, research demonstrated that the targeted knockout of *StCHL1* using CRISPR/Cas9 technology conferred increased resistance to *P. infestans* in potato plants (Figure 2) [21].

*StDMR6-1* is another well-studied susceptibility gene that encodes salicylic acid 5-hydroxylase (*SA5H*), a member of the 2-oxoglutarate (2OG)- and Fe(II)-dependent dioxygenase family. This enzyme catalyzes the hydroxylation of SA at the C5 position, converting it to 2,5-dihydroxybenzoic acid (2,5-DHBA), thereby maintaining SA homeostasis [37]. By reducing SA accumulation, *StDMR6-1* suppresses immune responses and helps to balance plant growth and defense [82,83]. However, loss-of-function mutations in *StDMR6-1* have been shown to enhance resistance to *P. infestans* by increasing SA levels and activating defense pathways [84]. In 2021, the CRISPR/Cas9 genome editing system has been effectively used to target and modify key potato susceptibility genes, such as *StMLO1*, *HDS*, *AtTTM2*, *StDND1*, *StCHL1*, and *StDMR6-1*. While mutant lines of *StMLO1*, *HDS*, and *AtTTM2* remained wild-type susceptible to late blight, tetra-allelic deletion mutants of *StDND1*, *StCHL1*, and *StDMR6-1* showed significantly improved resistance to *P. infestans* [21]. More recently, *StDMR6-1* mutant lines were further evaluated for resistance to additional stresses, including bacterial common scab, salt stress, and drought stress [37]. These mutants exhibited reduced visible scab lesions, increased biomass under salt stress, higher survival rates under PEG-induced drought conditions, and enhanced adaptation via stomatal regulation. Notably, the quality and yield of the tubers under field conditions remained comparable to that of wild-type plants, highlighting the potential of CRISPR/Cas-mediated editing for conferring broad-spectrum stress resistance in potato (Figure 2).

TFs play a crucial role in the regulation of plant defense by acting as molecular mediators that sense stress signals and direct the expression of downstream defense-related genes. In potatoes, SA and ethylene are the main signaling pathways activated in response to biotic stresses. These pathways activate specific TFs that regulate defense responses by binding to the promoter regions of target genes. *ERF3*, a member of the ethylene response factor (ERF) family, functions as a transcriptional repressor in ethylene signaling pathways [85]. ERF proteins bind specifically to the GCC box of the promoter regions of stress-responsive genes and modulate their expression through the ERF-associated amphiphilic repression (EAR) motif, which is characterized by the conserved sequence (L/F)DLN(L/F)xP at the C-terminus of the polypeptide chain [86]. One of the primary targets of *StERF3* is the *Isochorismate Synthase 1* (*ICS1*) gene, a key enzyme in the SA biosynthetic pathway. *StERF3* binds to the *ICS1* promoter region, repressing its transcription and thereby reducing SA synthesis via the isochorismate pathway. SA plays a critical role in systemic acquired resistance (SAR) and defense against biotrophic pathogens, including *P. infestans* [25]. Razzaq et al. [31] successfully applied the CRISPR/Cas9 system to the *Ethylene Responsive Transcription Factor* (*StERF3*) gene in the potato cultivar Lady Rosetta. The knockout lines showed a significantly higher relative expression of SA-mediated marker genes, such as *StPR1* (pathogenesis-related protein) and *StNPR1* (non-expressor of pathogenesis-related protein) (Figure 2) [76].

Calmodulin-binding transcription activators (CAMTAs), also known as signal responsive proteins (SRs) are a family of TFs characterized by the presence of calmodulin (CaM) binding sites within their structural domains. Recent studies have shown that members of the CAMTA/SRs are involved in plant hormone signal transduction pathways and play critical regulatory roles in plant responses to abiotic and biotic stresses. The potato *signal response 4* (*SR4*) gene, a homolog of Arabidopsis CAMTA3, plays a key role in modulating plant immunity. Under non-stress conditions, *SR4*/CAMTA3 binds to the promoters of key defense-related genes, such as *Enhanced Disease Susceptibility* 1 (*EDS1*) and *Non-race-specific Disease Resistance 1* (*NDR1*) and represses their transcription, thereby suppressing SA-mediated defense signaling [83,87]. This repression results in reduced SA accumulation, which weakens the plant’s immune response [88]. In potatoes, an influx of calcium ions upon pathogen attack activates calmodulin, which interacts with *StSR4*, leading to the de-repression of *EDS1* and *NDR1* [89]. However, the de-repression of these genes alone is insufficient to induce a fully effective immune response capable of halting disease progression. These findings suggest that *SR4*/CAMTA3 functions as a negative regulator of SA accumulation, making it a promising target for improving plant stress tolerance. The potato *StSR4* gene was edited using a CRISPR/Cas9 system delivered via the PEG-mediated protoplast transfection, with a maximum editing efficiency of 34%. The resulting mutants exhibited a significantly increased expression of *StEDS1* and *St*PAD4, resulting in high SA accumulation and an upregulation of *StPR1* expression. Activation of the SA biosynthesis pathway increased resistance to pathogens such as *P. infestans*. However, the increased SA levels also caused phenotypic changes in the mutants. The mutants exhibited a characteristic dwarf phenotype, with an increased number of branches and leaves per plant and a reduced number of leaves per plant (Figure 2) [25].

Plant immune responses are activated by the recognition of conserved microbial molecules, known as microbe- or pathogen-associated molecular patterns (MAMPs/PAMPs), which initiate pattern-triggered immunity (PTI). These responses are also activated by the detection of pathogen-secreted effectors, which trigger effector-triggered immunity (ETI). The potato *NPH3/RPT2-LIKE1* (*StNRL1*) gene encodes a regulatory protein that plays a key role in the plant’s immune response, especially during interactions with *P. infestans*. Studies on the transient overexpression of *NRL1* have shown that it suppresses INF1-mediated cell death and enhances *P. infestans* leaf colonization. This demonstrates that *NRL1* acts as a susceptibility factor that promotes late blight disease [90]. It was later discovered that the *P. infestans* effector Pi02860 uses the potato *StNRL1* protein as a susceptibility factor by promoting its ability to target a positive regulator of immunity *St*SWAP70 [91]. This finding suggests that *StNRL1* acts as a negative regulator of plant immunity by suppressing SWAP70-mediated defense pathways. CRISPR/Cas9-mediated genome editing was used to generate ten mutants in potato cultivar Agria, editing the exon regions of the *StNRL1* gene. Compared to the wild-type, the mutant line with a four-allelic knockdown of *StNRL1* gene showed a significant reduction of up to 90%, resulting in improved resistance to *P. infestans*. However, the mutant lines showed sensitivity to *Alternaria alternate*, suggesting that the *StNRL1* gene may play a role in resistance to early blight caused by *A. alternate* (Figure 2) [32].

Fungal–plant interactions are complex. During these interactions, each fungal species produces biomolecules that are secreted and either suppress or activate plant defense responses. Structural and secreted fungal metabolites are recognized by plant membrane receptors. *S. tuberosum* plasma membrane protein 1 (*StPM1*), which is encoded by a gene of the AWPM-19 like family, is involved in the defense response to *P. infestans*. Mechanistically, *StPM1* may interfere with receptor-like kinase (RLK) or pattern recognition receptor (PRR) complex formation at the plasma membrane, key components for pathogen-associated molecular pattern (PAMP) recognition and immune activation [92]. *StPM1* was identified as a susceptibility factor in potatoes in 2024 by Bi et al. (2024) [33] and was edited using CRISPR/Cas9 to improve resistance to *P. infestans*. Mutant lines showed milder disease symptoms and smaller lesions than wild types, while transgenic plants overexpressing *StPM1* became more susceptible to *P. infestans* and *P. capsici*, with reduced defense gene induction compared to wild types. Conversely, the mutants showed an increased expression of defense-related genes, including *StPR1*, *StPR5*, *StWRKY7*, and *StWRKY8* under infection (Figure 2) [33]. In 2025, Bi et al. (2025) [34] identified the *StDMP2* gene, which is an ER-localized member of the domain of unknown function 679 membrane protein (DMP) family (Figure 2). Its role in defense against *P. infestans* was confirmed by three types of experiments. First, StDMP2 was transiently expressed in *N. benthamiana* leaves, which were subsequently challenged with P. infestans. Compared to the green fluorescent protein (GFP) control, the expression of StDMP2 significantly reduced P. infestans infection symptoms in *N. benthamiana* leaves. Second, transgenic plants that constitutively express a hemagglutinin (HA)-tagged StDMP2 under the control of the 35S promoter were generated in both potato and *N. benthamiana*. The overexpression of *StDMP2* in four independent transgenic potato lines significantly reduced the *P. infestans* infection symptoms and enhanced resistance to infection. Third, mutated lines of the *StDMP2* gene were generated using the CRISPR/Cas9 system. The mutants exhibited more susceptibility than wild-type plants. Overall, these results indicate that *StDMP2* acts as a positive regulator in defense against Phytophthora pathogens.

### 3.2. Potato Virus Resistance

**Figure 3:**
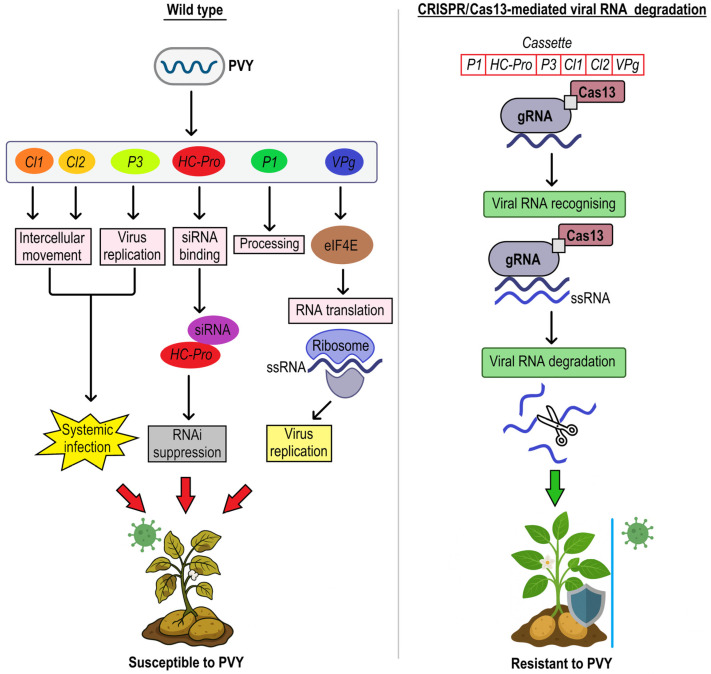
Molecular mechanism of *S. tuberosum eIF4E* and *Potato Virus Y* (PVY) genes *PI*, *HC-Pro*, *P3*, *CI1*, *CI2*, *VPg* and their role in resistance to PVY: In the wild-type potato (**left panel**), infected by PVY, genes encode key viral effectors, including *P1*, *HC-Pro*, *P3*, *CI*, and *VPg*. *VPg* recruits host *eIF4E* to initiate translation, *HC-Pro* suppresses RNA interference by sequestering siRNAs, facilitating unchecked viral replication and intercellular movement via P3N-PIPO (P3) and CI (*CI1*, *CI2*). This leads to systemic infection and visible disease symptoms. In contrast, the CRISPR/Cas-edited plant (**right panel**) expresses the multiple targeted guide RNAs from six key genes that specifically recognize and degrade PVY RNA upon infection, preventing the synthesis of viral proteins and halting replication.

Potato yield and quality depend on the rate of its infection by a number of viruses and viroids. Potato virus Y (PVY), a member of the *Potyviridae* family, is currently considered the most dangerous virus. PVY possesses a single-stranded, positive-sense RNA genome encoding a polyprotein, which is subsequently cleaved into proteins essential for its life cycle. Among these, the P1 protein functions as a serine protease that initiates processing of the viral polyprotein through autocatalytic cleavage. Following P1, the helper component proteinase (HC-Pro) is released; this multifunctional protein facilitates aphid-mediated transmission, suppresses RNA silencing, a key plant defense mechanism, and assists in viral replication by binding small interfering RNAs (siRNAs) and preventing their incorporation into the RNA-induced silencing complex (RISC). The P3 protein, characterized by two hydrophobic domains, is involved in viral replication, systemic infection, pathogenicity, and movement. A translational frameshift within the P3 cistron generates P3N-PIPO, which localizes to plasmodesmata and cooperates with the cylindrical inclusion (CI) protein to mediate cell-to-cell movement of the virus. The CI protein, which forms distinctive pinwheel-shaped cytoplasmic inclusions, exhibits RNA helicase and ATPase activities, underscoring its role in viral RNA replication and intercellular movement. In addition, the viral genome-linked protein (VPg), an intrinsically disordered protein, is covalently bound to the 5′-end of the viral RNA and interacts with the host eukaryotic initiation factor 4E (*eIF4E*), facilitating translation initiation and contributing to viral infectivity (Figure 3) [93].

The strain groups PVY(O), PVY(C), and PVY(N) are well established for the isolates infecting potatoes in the field. The primary symptoms of PVY^O^ and PVY^C^ are leaf mottling, wrinkling, and plant dwarfing. The PVY^N^ strain causes a non-necrotic mosaic on this host. Some PVY^N^ isolates cause potato tuber necrosis [94], and are referred to as PVY^NTN^. In efforts to control PVY infections in potato (*S. tuberosum*), the CRISPR/Cas13 system has emerged as a promising tool. Unlike DNA-targeting CRISPR/Cas variants, Cas13 specifically targets and degrades RNA molecules, enabling the direct cleavage of viral RNA genomes without altering the host plant DNA. CRISPR-Cas13 was used to target several genes in the PVY^N^, PVY^O^, and PVY^NTN^ strains by designing multiple guide RNAs (gRNAs) that are complementary to critical regions of the PVY genome, such as those encoding *PI*, *HC-Pro*, *P3*, *CI1*, *CI2*, and *VPg.* Three transgenic lines were obtained, and these lines showed a high expression of the Cas13a/sgRNA, resulting in resistance to several PVY strains [35]. This approach highlights the potential of CRISPR/Cas13-mediated RNA interference as an effective strategy for enhancing viral resistance in crops. Zhan et al. [23] developed another study demonstrating the consistent resistance to multiple PVY strains by targeting four different genes (*P3*, *CI*, *Nib*, and *CP*) using the CRISPR/Cas13a system [18]. The mutants expressing multiple gRNAs showed similar levels of PVY accumulation and reduced symptoms compared to those expressing only one gRNA. Despite the different levels of gRNA expression, the plants showed consistent resistance to multiple PVY strains. This result suggests that the number of gRNAs and their expression levels do not significantly affect the efficacy of viral interference (Figure 3).

Transcriptional regulation by TFs is critical for the establishment of plant defense and related activities during viral infection, making TFs promising targets for CRISPR/Cas-mediated improvement of resistance in potato. In potatoes, *eIF4E* is a critical host factor in the PVY infection cycle. The virus uses the host’s *eIF4E* to facilitate the translation of its RNA genome, which is essential for viral replication and systemic movement within the plant. Specifically, the PVY VPg (viral genome-linked protein) interacts directly with *eIF4E*, allowing the recruitment of the host translation machinery for viral protein synthesis. Mutations or deletions in *eIF4E* can disrupt this interaction, thereby limiting viral replication and movement (Figure 3) [95].

CRISPR/Cas9 was used to target and disrupt the *eIF4E* gene in potatoes to increase resistance to PVY. Knocking out *eIF4E* significantly reduced PVY accumulation and conferred increased resistance to the virus [96]. In the potato variety Kruda, the conserved homozygous region of *eIF4E* was edited using CRISPR/Cas9, resulting in insertion, deletion, point mutation, SNPs, and conversion events with ~15% editing efficiency. Consequently, all mutant lines showed a gradual decrease in virus titer and exhibited resistance at 60 days after PVY inoculation [36].

### 3.3. CRISPR/Cas System to Improve Nutritional Value of Potato

**Figure 4:**
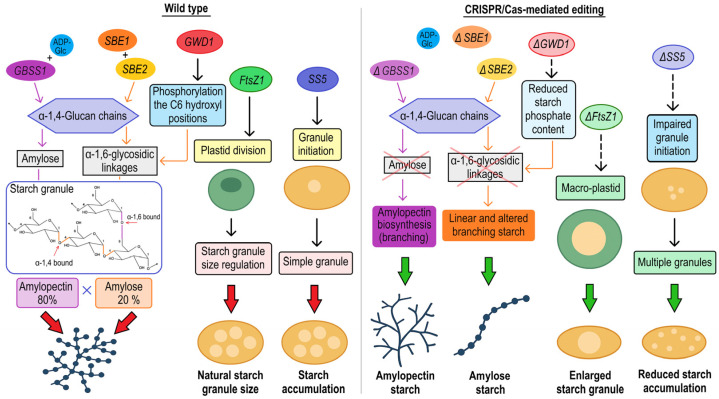
Molecular mechanism of *GBSS1*, *SBE1*, *SBE2*, *SS5*, *GWD1*, and *FtsZ1* genes and their role in tuber characteristics: In wild-type potato (**left panel**) expressing granule-bound starch synthase 1 (*GBSS1*), which catalyzes the elongation of linear α-1,4-glucan chains to facilitate amylose production, while starch branching enzymes *SBE1* and *SBE2* introduce α-1,6 linkages to form the branched amylopectin structure. *SS5* functions as a non-catalytic regulator of the granule initiation complex in amyloplasts, ensuring the spatial restriction of nucleation events and the formation of single, uniformly sized granules. *GWD1* phosphorylates the C6 hydroxyl of glucans, modulating granule structure and hydration, while *FtsZ1* governs plastid division and plastid size, which directly determines starch granule dimensions. CRISPR/Cas9-mediated knockout (**right panel**) of *GBSS1* disrupts amylose synthesis, resulting in amylopectin-rich starch with altered gelatinization properties. Simultaneous targeting of *SBE1* and *SBE2* modifies amylopectin architecture, increasing apparent amylose content and producing starch with improved industrial utility. CRISPR/Cas9 knockout of *StSS5* leads to excessive granule initiation, resulting in multiple small or compound granules and reduced starch yield. Loss of *GWD1* function reduces phosphate content in starch, elevates amylose levels, and alters thermal and viscosity properties of tubers, while also delaying tuber initiation. Genome editing of *FtsZ1* produces enlarged plastids (“macro-plastids”) that accumulate larger starch granules, significantly increasing final paste viscosity without negatively affecting tuber yield.

Starch is the most important component of potato tubers, and its structure and composition play a key role in its utilization, processing, and storage. The characteristics of potato starch are modified by the composition of amylose and amylopectin. *Granule-bound starch synthase I* (*GBSS1*) is essential for amylose biosynthesis by catalyzing α-1,4-glucan chain elongation with ADP-glucose. *GBSS1* activity is regulated by phosphorylation, which modulates its binding to starch granules and influences its catalytic efficiency [97]. Structural studies suggest that the *GBSS1* has a glycosyltransferase domain that contains a conserved KTGGL motif, which interacts with ADP-Glc and facilitates the transfer reaction [98]. *GBSS1* is encoded by a single gene and has often served as a model target for genome editing approaches using CRISPR/Cas9 technology. In 2021, CRISPR/Cas9-mediated mutagenesis was used to knock out the *GBSS1* gene in the tetraploid potato cultivar ‘Yukon Gold’ to produce amylose-free starch in tubers [38]. Introducing targeted mutations in all four *GBSS1* alleles completely disrupted amylose synthesis, resulting in a starch composition that is predominantly amylopectin [38]. In a subsequent study, Abeuova et al. [39], modified local cultivars Astanalyk, Tokhtar, and Aksor to obtain an amylose-free phenotype by editing three regions of exon I in the *StGBSS* gene using the CRISPR/Cas9 system. Local potato cultivars were selected for transformation because they were confirmed to have a high frequency of direct shoot regeneration ability [99]. Tetra-allelic mutant lines yielded amylopectin starch due to a frameshift mutation including insertions, deletions, and substitutions (Figure 4).

Starch branching enzymes (SBEs) play a critical role in the biosynthesis of potato amylopectin by introducing α-1,6-glycosidic linkages into linear glucan chains. The two primary isoforms, *SBE1* and *SBE2*, have distinct functions: *SBE1* acts on longer chains, whereas *SBE2* acts on shorter ones. Modifying the activity of these enzymes, particularly by downregulating *SBE2*, has been shown to alter amylopectin architecture and increase apparent amylose content [100,101]. In a recent study, Zhao et al. [26] used DNA-free CRISPR/Cas9 mutagenesis with ribonucleoprotein (RNP) transfection to edit the *SBE1* and *SBE2* genes in tetraploid potatoes, achieving mutation frequencies of up to 72%. Similarly, mutant lines of the SBE3 gene from the Sayaka cultivar were generated by Takeuchi, et al. [40] using CRISPR/Cas9 in combination with the translation enhancer dMac3, achieving a mutagenesis efficiency of 8%. Together, these studies demonstrate the precision and potential of genome editing technologies for metabolic engineering and improving starch quality in potatoes (Figure 4).

In potato, starch Synthase 5 (*StSS5*) functions as a regulatory component of the granule initiation complex in amyloplasts (Figure 4). This complex is essential for spatially restricting starch nucleation events and ensuring the formation of simple, single-centered starch granules. Although *StSS5* lacks canonical glucosyltransferase activity, it likely interacts with primer structures or scaffolding elements within the initiation zone to coordinate the recruitment or spatial arrangement of active starch synthases. This non-enzymatic role is consistent with findings in *Arabidopsis thaliana*, where the orthologous SS5 regulates the number of granules rather than their elongation. In potato tubers, *StSS5* ensures proper plastidial architecture and granule morphology by balancing granule size distribution and preventing the formation of compound granules. Hu et al., 2025 [42] aimed to understand the role of *StSS5*, which exhibits distinct starch granule initiation patterns in leaf chloroplasts and tuber amyloplasts. In the leaves of the ss5 mutants, fewer starch granules formed in the chloroplasts, and starch breakdown during the night decreased. In contrast, the ss5 mutation in tubers led to an increased number of initiation sites for starch granules, resulting in compound granules and a higher quantity of smaller starch granules.

**Figure 5:**
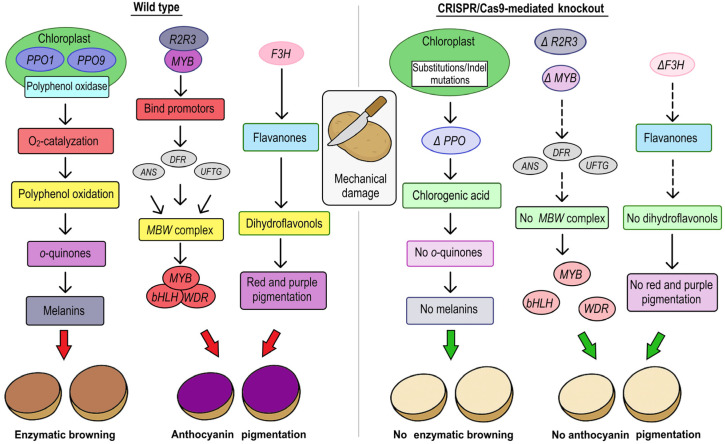
Molecular mechanism of the *PPO*, *R2R3-MYB*, *F3H* genes and their role in oxidative stress and secondary metabolism: In wild-type potato tubers (**left panel**), PPO enzymes encoded by the *StPPO* gene are produced to catalyze the oxidation of chlorogenic acid to o-quinones upon tissue damage. These reactive intermediates polymerize into melanins, resulting in visible tissue browning and reduced tuber quality. Concurrently, two tandem *R2R3*-*MYB* transcription factors regulate the anthocyanin biosynthetic pathway by binding to the promoters of key structural genes (*DFR*, *ANS*, *UFGT*) and recruiting bHLH and WDR cofactors to form the MBW complex, promoting transcription and anthocyanin accumulation in the tuber flesh. *F3H* catalyzes an essential early step in this pathway by converting flavanones to dihydroflavonols, enabling flux toward anthocyanin biosynthesis and contributing to red and purple pigmentation. CRISPR/Cas9-mediated knockout of *StPPO* genes (**right panel**) disrupts PPO activity, preventing quinone formation and subsequent melanin production, thereby reducing tissue browning. Simultaneously, knockout of both tandem *R2R3*-*MYB* genes abolishes MBW complex assembly and downstream transcriptional activation of anthocyanin pathway genes. As a result, anthocyanin synthesis is silenced, leading to unpigmented tuber flesh. Complete CRISPR/Cas9 knockout of all *F3H* alleles likewise terminates anthocyanin biosynthesis by blocking dihydroflavonol production, leading to stable, multigenerational tubers with yellow, unpigmented flesh, while preserving yield and general performance.

Tandem R2R3 MYB transcription factors in potato tuber flesh form a cooperative regulatory module that controls anthocyanin biosynthesis (Figure 5) [102]. These proteins contain two conserved MYB DNA-binding repeats and bind preferentially to the promoters of late-pathway anthocyanin genes, such as *DFR*, *ANS*, and *UFGT*. This binding recruits transcriptional co-activators, including bHLH transcription factors, which assemble the MBW (*MYB-bHLH-WDR*) complex that drives pigment production [103]. The biosynthesis and accumulation of anthocyanins not only determine the color of potato tuber flesh, but also influence nutritional quality and act as antioxidants under abiotic and biotic stresses. The CRISPR/Cas9 system, using *Agrobacterium*-mediated transformation, was employed to investigate the regulatory roles of tandem *R2R3 MYB* genes, including *StMYB200* and *StMYB210*, in anthocyanin accumulation in potato tuber flesh. This revealed that both genes activate and interact with the bHLH TF gene *StbHLH1.* Additionally, promoter analysis of *StMYB210* across mutants indicated that insertion events are associated with variation in tuber flesh color [43].

α-Glucan water dikinase 1 (*GWD1*) phosphorylates the C6-hydroxyl positions of starch glucans within potato amyloplasts (Figure 4). This modification is crucial for modulating starch granule hydration, structural organization, and the activity of enzymes involved in starch degradation. The loss of *GWD1* significantly reduces starch phosphate content, resulting in higher apparent amylose levels, a decreased gelatinization temperature, and a lowered peak viscosity. These phenotypes support the role of phosphate groups in stabilizing the starch structure and retaining moisture. These structural alterations also contribute to reduced tissue syneresis during freeze–thaw cycles, which is beneficial for industrial starch applications. Transgenic lines of cv. Sayaka, generated using the CRISPR/Cas9 system, exhibited targeted deletions, insertions, and substitution in the *GWD1* gene. Compared to the wild type, these mutant lines showed decreased phosphorus content, significantly less water loss, and a higher amylose content [44].

*FtsZ1*, a tubulin-like GTPase, is essential for plastid division in potatoes (Figure 4). It coordinates the formation of the plastid constriction ring, which determines the number and size plastids. In edited potato tuber cells, reduced *FtsZ1* expression disrupts plastid fission, resulting in fewer, enlarged “macro-plastids”. These larger plastids contain proportionally larger starch granules, suggesting that plastid size directly impacts starch granule morphology. The increase in starch granule size is functionally correlated with altered physicochemical properties; starch paste from edited lines exhibited approximately double the final viscosity, reflecting the influence of granule architecture on industrial performance. Desiree cultivar lines mutated using the CRISPR/Cas9 system showed reduced *FtsZ1* gene expression and increased starch granule size without affecting nutritional quality [54].

**Figure 6:**
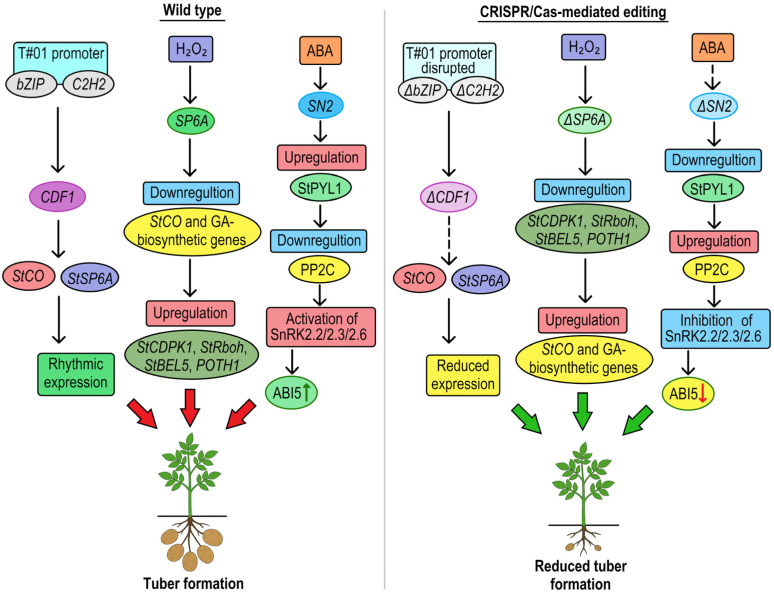
Molecular mechanisms of *SN2*, *SP6A*, *CDF1* genes and their role in the regulation of potato tuberization. In wild-type potato (**left panel**), *SN2* functions as a positive regulator of abscisic acid (ABA) signaling by upregulating the ABA receptor *StPYL1* and repressing *PP2C*, which in turn, activates *SnRK2.2/2.3/2.6* kinases and enhances *ABI5*-mediated transcription, promoting tuber initiation. *SP6A* encodes a mobile FT-like protein that integrates H_2_O_2_ signaling with photoperiod and sucrose-responsive pathways. H_2_O_2_ accumulation induces *SP6A* expression, repressing *StCO*, and GA biosynthesis (*GA20ox1*), while activating *StBEL5*, *StCDPK1*, *POTH1*, and *StRboh* to drive stolon swelling and tuber formation. *CDF1*, under short-day conditions, is rhythmically expressed from a 288 bp light- and clock-responsive promoter and represses *StCO*, thereby derepressing *SP6A* and activating the tuberization program. Under long-day conditions, *CDF1* is destabilized by *StFKF1* and *StGI*, preventing premature tuber formation. CRISPR/Cas9-mediated knockout of *StSN2* (**right panel**) diminishes ABA signal amplification, suppressing ABI5 activation and leading to reduced tuber formation. Disruption of *SP6A* abolishes H_2_O_2_ responsiveness, silencing downstream signaling and inhibiting tuber induction. Targeted deletion of cis-elements within the *CDF1* promoter reduces its photoperiod-regulated expression amplitude, delaying tuber initiation by ~5–6 days and moderately reducing plant biomass, while maintaining its rhythmic expression pattern.

*StCDF1* encodes a DOF transcription factor whose circadian and photoperiod-regulated expression controls the timing of tuber formation in potato. Its transcription is governed by a core 288 bp promoter region (denoted T#01), which harbors light- and clock-responsive cis-regulatory elements (e.g., bZIP and C_2_H_2_ binding sites) that confer higher promoter activity under short days. This promoter mediates rhythmic *StCDF1* expression, peaking before dawn and driving the repression of CONSTANS (StCO), thereby derepressing *StSP6A* and initiating downstream tuberigen signaling. In long days, *StCDF1* is destabilized via interaction with *StFKF1* and StGI, preventing untimely tuber induction. Thus, *StCDF1* integrates environmental light cues via its promoter to modulate gene networks governing photoperiod-dependent tuberization. CRISPR/Cas9-editing line of the *StCDF1* gene was established (Figure 6), with two deletions in the core promoter. This line displayed a reduced expression levels and late tuberization under both long-day and short-day conditions [45].

*StSP6A* encodes the mobile tuberigen FT-like protein, which acts as a biochemical hub in stolons that integrate H_2_O_2_ signaling with the photoperiodic and sucrose-dependent tuberization pathway (Figure 6). Elevated H_2_O_2_ accumulation in the subapical region of stolons serves as a signaling cue that significantly induces *StSP6A* expression early in tuber initiation, likely through redox-sensitive transcriptional or post-translational mechanisms. This H_2_O_2_-mediated activation of *StSP6A* downregulates the repressor *StCO* and GA biosynthetic genes (e.g., *GA20ox1*), while upregulating downstream components including *StCDPK1*, *StRboh*, *StBEL5*, and *POTH1.* This establishes an integrated signaling cascade that promotes stolon swelling and tuber development. When Lei et al. (2023) [46] used CRISPR/Cas9 to disable the *StSP6A* gene in cultivars CIP-149 and CIP-178, the mutants showed a significant decrease in tuber formation, even when exposed to a different concentration of hydrogen peroxide. These results demonstrate that *StSP6A* plays a crucial role in tuber development that cannot be easily replaced or triggered by H_2_O_2_ signaling alone.

A functional analysis of *StSN2* revealed its role as a positive regulator of tuber formation by directly enhancing the ABA signaling cascade (Figure 6). The gene is transcriptionally activated by ABA via ABRE motifs in its promoter. In turn, it amplifies ABA signaling by upregulating the receptor *StPYL1* and suppressing *PP2C* expression. This relieves inhibition on *SnRK2* kinases. This results in the increased expression and activation of *SnRK2.2/2.3/2.6*, which leads to elevated levels of the transcription factor *ABI5*, a key effector of ABA-responsive gene expression. Liu et al. studied the modulation of StSN2 expression in potatoes using CRISPR/Cas9, revealing that *StSN2* promotes tuber formation by enhancing ABA signaling through the upregulation of the *StPYL1*, *StSnRK2.2/2.3/2.6*, and *StABI5* genes. This causes delayed stolon development and a 20–30% reduction in yield [48].

**Figure 7:**
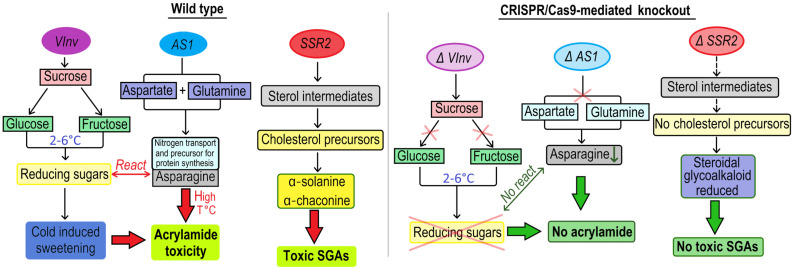
Molecular mechanism of *VInv*, *AS1*, *SSR2* genes and their role in tuber characteristics: In wild-type potato (**left panel**) expressing *VInv*, which hydrolyzes sucrose to glucose and fructose during cold storage, leading to cold-induced sweetening (CIS) and, upon frying, increased acrylamide formation via Maillard reactions. At the same time, *AS1* catalyzes the biosynthesis of asparagine, a key acrylamide precursor. *SSR2* encodes the sterol side-chain reductase 2 enzyme that catalyzes the formation of cholesterol precursors for steroidal glycoalkaloid (SGA) biosynthesis. Its activity determines the metabolic flux toward α-solanine and α-chaconine, major toxic SGAs that must be tightly regulated due to food safety concerns. CRISPR/Cas9-mediated knockouts of *VInv* and *AS1* (**right panel**) significantly reduce sugar and free asparagine levels, respectively, mitigating browning and acrylamide accumulation during processing. CRISPR/Cas9-mediated disruption of *SSR2* dramatically reduces total SGA content in tuber tissues, highlighting its utility as a target gene for minimizing glycoalkaloid toxicity.

The *StSSR2* gene encodes the sterol side-chain reductase 2 enzyme, which catalyzes the conversion of sterol intermediates into cholesterol precursors that are essential for the biosynthesis of steroidal glycoalkaloid (SGA) in potatoes (Figure 7). This enzymatic step determines the flux of toxic SGAs, such as α-solanine and α-chaconine, whose accumulation is tightly regulated due to their anti-nutritional properties. *StSSR2* modulates sterol pathway output and governs the pool of SGA precursors within tuber tissues. CRISPR/Cas9 editing of the *StSSR2* gene in potatoes produced 64 mutant potato lines with a 46% mutation efficiency, leading to a significant reduction in steroidal glycoalkaloid content [49].

In improving potato traits, CRISPR/Cas gene editing technology is used not only to alter the nutritional value of tubers, but also to alter their color. For example, *StF3H* (*flavanone 3-hydroxylase*) catalyzes the conversion of flavanones to dihydroflavonols, which is a pivotal biochemical step in the anthocyanin biosynthetic pathway that is responsible for the red and purple pigmentation of potato tubers (Figure 7). Despite the presence of multiple allelic loci, the accumulation of anthocyanins is only abolished by the complete knockout of all four *F3H* alleles, which demonstrates the dominant biochemical effect of even a single functional allele. Protoplast-derived potatoes engineered using CRISPR/Cas9 to target the *F3H* gene exhibited disrupted anthocyanin biosynthesis. Consequently, a multigenerational phenotypic analyses across three successive tuber generations revealed stable knockouts in one to four alleles. These changes led to variation in skin pigmentation, temperature-dependent tuber phenotypes, and instances of somaclonal variation. These findings underscore the importance of critical developmental and environmental interactions that would have gone unnoticed without conducting extended generational trials [47].

### 3.4. CRISPR/Cas System to Reduce of Postharvest Factors Affecting Potatoes

The economic efficiency of a potato variety is determined by its yield and its ability to maintain quality during storage. After harvest, tubers are stored at low temperatures (2–4 °C) for up to several months. However, long-term storage of the potato crop at low temperatures, which ensures good storage of the tubers, leads to significant physiological changes in the tubers. These changes make the tubers unsuitable for processing into products such as crisps and French fries, because heat treatment of such tubers (boiling or frying) causes the reducing sugars to interact with α-amino acids to form acrylamide, a carcinogen. Currently, many studies that have demonstrated the role of vacuolar invertase in cold saccharification in potatoes, which catalyzes the irreversible hydrolysis of sucrose [104,105,106]. This enzymatic process plays a central role in carbohydrate metabolism, especially under cold storage conditions. There, the accumulation of reducing sugars (glucose and fructose) leads to a phenomenon known as cold-induced sweetening (CIS). Invertases, also called soluble acid invertases, are characterized by an acidic pH optimum (pH 5.0–5.5) and are localized in the vacuole. In potatoes, vacuolar acid invertases are encoded by the *StInv* gene family. Vacuolar invertases are expressed in tubers, leaves, roots, and stems (Figure 7). In 2022, CRISPR/Cas9-mediated knockdown of the *Vacuolar Invertase* (*VInv*) gene was performed using two sgRNAs in the potato cultivar AGB Purple. These genetic modifications resulted in a significant reduction in *VInv* gene expression by 90- to 99-fold, leading to a clear difference in the amount of reducing sugars in the edited lines compared to the control type [41]. In 2024, Zhu et al. discovered that the expression of the *VInv* gene in response to cold is regulated by a 200 bp enhancer located in the second intron. CRISPR/Cas9-mediated editing of this region resulted in a 54% reduction in *VInv* expression in the mutated lines [51] (Figure 7).

It is known that during high-temperature cooking, asparagine reacts with reducing sugars to form acrylamide [107]. *Asparagine synthetase 1* (*AS1*) is an enzyme responsible for the biosynthesis of asparagine from aspartate and glutamine [108]. Therefore, to prevent a sweet taste in frozen potatoes and to reduce the activity of genes responsible for acrylamide formation, CRISPR/Cas9 technology was used to target the *VInv* and *AS1* genes in potato cultivars. After cold storage, the tubers of full knockout mutant lines exhibited reduced sugar concentrations and 85% less acrylamide (Figure 7) [50].

Enzymatic browning is one of the most important factors that limits the postharvest life of potatoes. The main enzyme involved in the oxidation of phenolic compounds is polyphenol oxidase (PPO) which plays a dual role in potatoes. PPO contributes to enzymatic browning after tissue damage and participates in defense responses through an oxidative burst. In potatoes, multiple *StPPO* genes (*StuPPO1*-*StuPPO9*) are distributed across different chromosomes and encode proteins that share conserved domains characteristic of PPOs, including the tyrosinase domain (pfam00264), the PPO1_DWL domain (pfam12142), and the PPO1_KFDV domain (pfam12143) [109]. Tissue browning occurs when PPOs oxidize polyphenols, mainly chlorogenic acid to quinones, which then polymerize into melanins [110,111]. Enzymatic browning results in undesirable color changes and can lead to a loss of nutritional quality and off-flavors. To mitigate this, CRISPR/Cas9 was used to target *StPPO* genes by introducing indels that disrupt gene function. Edited lines show reduced PPO activity and reduced quinone formation, and improved resistance to browning. However, the results of this study revealed off-target activity caused by somatic mosaicism. Two canonical off-target sites were identified on chromosome 8 of heterozygous diploid lines. The first off-target site overlapped with the CDS of *StPPO2-2*, and the second off-target site overlapped with the 5′ UTR of the following gene model *RHC08H2G1680.2*. and *RHC08H2G1680.2* [52]. Massa et al. (2025) [53] used CRISPR/Cas9 and protoplast transfection to edit the *VInv* and *PPO2* genes. This resulted in improved chip quality and reduced browning while largely preserving tuber traits in the Atlantic and Spunta lines. These results inform the implementation and risk assessment of genome editing in commercially valuable food crops traits (Figure 5).

## 4. Conclusions and Future Perspectives

We provided an overview of how potatoes respond to various environmental stresses, such as drought, salinity, and fungal and viral infections. We also discussed the impact of these stresses on plant growth, productivity, and postharvest quality. Additionally, we discussed the molecular mechanisms underlying these stress responses and highlighted recent research on CRISPR/Cas-mediated genome editing aimed at enhancing stress tolerance and improving the nutritional value of potato plants.

As mentioned in our previous review [112], between 2015 and 2021, genome editing in potatoes using the CRISPR/Cas9 system primarily targeted marker genes and was performed in model cultivars, such as Desiree and Atlantic. More recent studies have expanded these efforts to include commercial varieties and target multiple genes to achieve broader stress resistance [28,29,31,39]. A major constraint in this area has been the low embryogenic competency of many local cultivars in tissue culture systems. Since 2021, however, an increasing number of studies have successfully applied CRISPR/Cas editing to local and commercial cultivars and various clones [33,52].

Despite these advances, the efficiency of editing is still limited by several technical factors, which has prompted researchers to develop improvement strategies. For example, optimizing promoters, vectors, and translation enhancers has been shown to increase Cas9 expression and editing efficiency. Rather et al. [27] achieved a 95% editing frequency of in regenerated calli using linearized DNA constructs with UBIQUITIN10 promoters and optimized kanamycin selection via NEOMYCIN PHOSPHOTRANSFERASE2 (NPT2) and transient expression of BABY BOOM (BBM). Lee, Kang [55] developed a transgene-free genome editing method that uses a Potato Virus X (PVX) vector to deliver Cas9 and sgRNAs. They found that heat treatment increased editing efficiency from 56% to 76%. Similarly, Takeuchi et al. [40] used a CRISPR/Cas9 system enhanced with the translation booster dMac3 and achieved a mutagenesis efficiency of 8%. Editing efficiency can vary depending on whether the CRISPR system targets the 5′ or 3′ end of a gene, because different regions may differ in accessibility or sequence. This affects how effectively the guide RNA directs the Cas protein to induce edits. To study these variations, the *GWD1* and *DMR6-1* genes were edited using CRISPR/Cas system with gRNAs designed from different target exon comprising the 5′ and the 3′ ends. Mutant lines of the *GWD1* gene with regions targeted at the 5′ end resulted significantly higher editing efficiency than lines with regions targeted at the 3′ end. In mutant lines of the *DMR6-1* gene, the editing efficiency was more balanced between the 5′ and 3′ ends [56]. In the study by Lukan et al. (2022), miRNA (*MIR160a*, *MIR160b*, *and MIR390a*) editing using CRISPR/Cas9 was found to be an effective method for adjusting miRNA expression levels, achieving high editing efficiency [57]. Looking ahead, developing cultivar-independent transformation systems, such as de novo meristem induction or the use of morphogenic regulators, like BABY BOOM and WUSCHEL, holds significant promise for improving the efficiency of transformation and editing across a broader range of potato genotypes.

The molecular mechanisms underlying the function of negative regulatory genes in the stress response are an active area of research. Based on studies of other plant species, it is likely that these genes contribute to the biosynthesis or modification of compounds involved in defense and stress responses. Further studies are needed to elucidate the specific proteins and pathways associated with these genes, their regulatory mechanisms, and their effects on potato physiology.

Although the CRISPR/Cas9 system has been successfully used to edit genes that negatively regulate drought tolerance in several plant species [113,114,115], resulting in improved drought resistance, there is still very limited information and research on the applying this approach to potatoes. Further studies are needed to identify and validate negative regulators of drought tolerance in potatoes and to assess their potential as genome-editing targets for improving drought tolerance. Additionally, efforts must be made to improve editing precision, minimize off-target effects, and ensure regulatory compliance. Combining genome editing with approaches, such as GWAS, transcriptomics, and marker-assisted selection, will also be critical for developing potato varieties adapted to different agroecological environments. Ultimately, deepening our understanding of the molecular mechanisms underlying stress tolerance and applying cutting-edge biotechnologies will be essential to ensuring sustainable potato production in the face of ongoing climate change. Overall, CRISPR/Cas9 shows promise in developing resilient, and high-quality potato cultivars, but further refinement is necessary for its safe and effective application.

## Figures and Tables

**Table 1:** Research studies on application of CRISPR/Cas-mediated editing technology in potato (*S. tuberosum*) from 2021 to 2025.

Cultivar	Method of Delivery	Technology	Promoters	Transgenic or DNA-Free Genome Editing	Target Gene (s)	Trait Associated with the Genes	Type of Mutation	Results	Reference
**CRISPR/Cas system for the enhancement of tolerance abiotic stress**									
Cv.CIP 149	Agrobacterium-mediated transformation (*A. rhizogenes*); pKESE401 vector and pCBC-DT1T2 intermediate vector	CRISPR/Cas9 system	U6 promoter	transgenic	*StFMO* GS-OX-Like3 (*StLike3*)	tolerance to salt stress	chimeras, deletion, insertion and replacement	Significantly increased mutation efficiency under appropriate NaCl and mannitol concentrations; no off-target effects were found, but root regeneration was inhibited.	[28]
Cv. Kufri jyoti	Agrobacterium-mediated transformation (*A. rhizogenes*); pHSE401 vector	CRISPR/Cas9 system	AtU6 promoter	transgenic	*StbHLH47*	iron regulation	deletion	Showed reduced ferric chelate reductase (FCR) activity; but increased expression of iron uptake-related genes, resulting in significantly higher Fe(II) accumulation in tuber tissues; changes in phenotype with short and thin trichomes on stem.	[29]
Cv. Li Shu 6	Agrobacterium-mediated transformation; pBWA(V)KS vector	CRISPR/Cas9 system	35S promoter	transgenic	Deeper Rooting 1 (*StDRO2*)	regulation of the root growth	deletion and insertion	Mutant lines exhibited higher plant height, longer root length, smaller root growth angle, and increased tuber weight than the wild-type.	[30]
**CRISPR/Cas system for the enhancement of tolerance biotic stress**									
Cv. Desiree and King Edward	*Agrobacterium-*mediated transformation; Csy4 multi-gRNA vector	CRISPR/Cas9 system	-	transgenic	*StDND1* and *StCHL1*, *StDMR6-1*, *StDMR6-2*	tolerance to late blight pathogen (*P. infestans*)	deletion (indel)	*St*DND1, *StCHL1*, and *StDMR6-1* mutants showed increased resistance to late blight.	[21]
Cv. Lady Rosetta	*Agrobacterium-*mediated transformation; pYLCRISPR/Cas9Pubi-B binary vector	CRISPR/Cas9 system	ubiquitin promoter derived from *Oryza sativa* (OsU6a)	transgenic	ERF transcription factor (*StERF3*)	tolerance to late blight pathogen (*P. infestans*)	deletion (indel)	Improved resistance to *P. infestans* and relatively high expression of *StPR1* and *St*NPR1.	[31]
Cv. Desiree	PEG-mediated protoplast transfection of ribonucleoprotein (RNPs)	CRISPR/Cas9 system	*StSR4* binds to the promoters of *EDS1* and *NDR1*	DNA-free genome editing	Signal Responsive 4 (*StSR4*)	tolerance to late blight pathogen (*P. infestans*)	insertion and deletion (indel)	Improved resistance to *P. infestans* and the expression of *StEDS1*, *St*PAD4, and *StPR1*; resulted in stunted growth and a dwarf phenotype.	[25]
Cv. Agria	*Agrobacterium-*mediated transformation	CRISPR/Cas9 system	U6-26 promotor	transgenic	NPH3/ RPT2-LIKE1 protein (*StNRL1*)	tolerance to late blight pathogen (*P. infestans*) and early blight (*A. alternata*)	deletion (indel)	Improved resistance to *P. infestans* and sensitivity to *A. alternate.*	[32]
Phureja S15-65 clone	*Agrobacterium* mediated transformation; pTC212, pTC241, and pCGS752 vectors	CRISPR/Cas9 system	35S promoter	transgenic	Plasma membrane protein 1 (*StPM1*)	tolerance to late blight pathogen (*P. infestans*)	deletions and the consequent frameshift mutations	Milder disease symptoms and smaller lesions than wild types; overexpressing *StPM1* and more susceptible to *P. infestans* and *P. capsici.*	[33]
Phureja S15-65 clone	*Agrobacterium* mediated transformation; pCAMBIA-1300-35S vectors	CRISPR/Cas9 system	35S promoter	transgenic	DOMAIN OF UNKNOWN FUNCTION 679 membrane protein (DMP) (*StDMP2*)	tolerance to late blight pathogen (*P. infestans*)	-	First, subsequently challenged with *P. infestans*. Second, overexpression of *StDMP2* was significantly reduced the *P. infestans* infection symptoms and enhanced resistance to infection. Third, the mutants exhibited more susceptibility to *P. infestans* than wild-type plants.	[34]
Cv. Kruda	*Agrobacterium* mediated transformation; pK2GW7-pCas13a vector and PTZ57R^kana^ vector	CRISPR/Cas13a system	Arabidopsis U6 promoter, 35S promoter	transgenic	six genes (*PI*, *HC-Pro*, *P3*, *CI1*, *CI2*, and *VPg*) of potato virus Y (PVY)	tolerance to broad-spectrum resistance	-	High expression of Cas13a/sgRNA resulting in resistance to PVY strains.	[35,36]
Cv. Kruda	*Agrobacterium-*mediated transformation; binary expression vector pK2GW7	CRISPR/Cas9 system	35S promoter	transgenic	eukaryotic translation initiation factor (*eIF4E*)	tolerance to potato virus Y resistance	insertion, deletion, and point mutation, and conversion events	Exhibited a gradual decrease in virus titer and resistance to PVY.	[36]
Cv. Desiree	*Agrobacterium* mediated transformation	CRISPR/Cas13a system	*Arabidopsis* U6 promoter	transgenic	four genes (*P3*, *CI*, *Nib*, and *CP*) of PVY	tolerance to potato virus Y resistance	-	Similar viral accumulation and reduced symptoms, consistent resistance to various PVY strains.	[23]
Cv. KingEdward	*Agrobacterium* mediated transformation; Csy4 multi-gRNA vector	CRISPR/Cas9 system	*-*	transgenic	Downy mildew Resistance 6 (*StDMR6-1*)	tolerance to bacterial scab, salt stress and drought stress	-	Exhibited fewer scab lesions, greater fresh weight and survival rates; improved tuber quality and faster adaptation in the field.	[37]
**CRISPR/Cas system for the enhancement of nutrient contents in potato**									
Cv.Yukon Gold	*Agrobacterium*-mediated transformation; pCGS752 (base binary vector),	CRISPR/Cas9 system	*-*	transgenic	Granule-bound starch synthase (*GBSSI*)	amylose-free starch	indels (insertions and deletions)	A reduction or complete elimination of amylose.	[38]
Cv. Astanalyk, Tokhtar, and Aksor	*Agrobacterium*-mediated transformation; pEn-Chimera, pMR203, pMR204, and pMR205 vectors	CRISPR/Cas9 system	AtU6 promoter	transgenic	Granule-bound starch synthase (*StGBSS*)	amylose-free starch	substitutions and indels	Resulting in an amylose-free phenotype.	[39]
Cv. Desiree	PEG-mediated protoplast transfection of ribonucleoprotein (RNPs)	CRISPR/Cas9 system	*-*	DNA-free genome editing	Starch branching enzyme (*SBE1* and *SBE2*)	amylopectin-free starch	deletion, insertion	Resulting in no amylopectin with no detectable branching and a high mutation frequency.	[26]
Cv. Sayaka	*Agrobacterium*-mediated transformation; pMR203, pMR204, and pMR205	CRISPR/dMac3-Cas9 system	AtU6-26 promoter	transgenic	Starch-branching enzyme (SBE) (*StSBE3*)	amylopectin- free starch	deletion, frameshift mutations	Resulting in 8% target efficiency and loss of function of SBE3, mutants grew normally, and yielded sufficient amounts of tubers.	[40]
Cv. AGB Purple	*Agrobacterium*-mediated transformation; pDES vector	CRISPR/Cas9 system	TA cloning kit dual Promoter (PCR^®^ II)	transgenic	Vacuolar invertase (*VInv*)	tolerance to cold-induced sweetening	indels, frame shift mutations	Resulting reducing sugars and editing efficiencies; no morphological variations were observed in the edited lines; however, there were notable differences in physical characteristics.	[41]
Cv. AC142	*Agrobacterium*-mediated transformation; pH7LIC-N-eGFP vector	CRISPR/Cas9 system	potato U6 promoter and CaMV 35S promoter	transgenic	Starch Synthase 5 (*StSS5*)	number and morphology of starch granules	deletion, insertion	In tubers, the *ss5* mutation increased starch granule initiation sites, producing compound and more small granules.	[42]
Clone 01–58	*Agrobacterium*-mediated transformation; pCAMBIA2300MGFPuv-sgRNACas vector	CRISPR/Cas9 system	StMYB210 promoter		Tandem R2R3 MYB genes (*StMYB200* and *StMYB210*)	regulation anthocyanin accumulation in tuber flesh	deletion, insertion	Both *StMYB200* and *StMYB210* activate the expression of the bHLH TF gene *StbHLH1* and interact with it to regulate anthocyanin biosynthesis. Analysis of the *StMYB210* promoter in various diploid potato accessions revealed that insertion events were associated with flesh color.	[43]
Cv. Sayaka	*Agrobacterium-*mediated transformation	CRISPR/Cas9 system	AtU6-26 promoter	transgenic	α-glucan water dikinase 1 gene (*GWD1*)	retaining moisture and stabilization of the starch structure	deletion, insertion, and substitution	The *gwd1* mutant tubers showed decreased phosphorus content, significantly less water loss, and a higher amylose content.	[44]
Cv. DM 1–3 516 R44	*Agrobacterium-*mediated transformation; *pKGWFS 7.0* vector	CRISPR/Cas9 system	35S promoter	transgenic	CYCLING DOF FACTOR 1 (*StCDF1*)	late tuberization	deletions	Displayed a reduced expression level, resulting in late tuberization under both long-day and short-day conditions.	[45]
Cvs. CIP-149 and CIP-178	*Agrobacterium*-mediated transformation	CRISPR/Cas9 system	GA20ox1 promoter	transgenic	Flowering locus T (FT)-like self-pruning 6A *(StSP6A)*	induction and for formation of potato tubers	deletions and insertions	The mutants showed a marked drop in tuber formation.	[46]
Cvs. Desirée and Nansen	PEG-mediated protoplast transfection of ribonucleoprotein (RNPs)	CRISPR/Cas9 system	-	DNA-free genome editing	flavanone 3-hydroxylase (*F3H)*	formation of the anthocyanidins	deletion	Observed changes in skin pigmentation, temperature-dependent tuber phenotypes, and instances of somaclonal variation.	[47]
Cv. Desirée	*Agrobacterium-*mediated transformation	CRISPR/Cas9 system	*StSN2* *promoter*	transgenic	Snf1-related protein kinase 2.2 (snrk2.2) or *StSN2*	regulation of tuber formation	deletions, Insertions, and SNPs	Mutation lines promote tuber formation by enhancing ABA signaling, specifically through upregulation of *StPYL1*, *StSnRK2.2/2.3/2.6*, and *StABI5* genes.	[48]
*Cv. Atlantic*	*Agrobacterium-*mediated transformation; VK005-*StSSR2 vector*	CRISPR/Cas9 system	AtU6 promoter		sterol side-chain reductase 2 enzyme *(StSSR2)*	reduction in steroidal glycoalkaloid content	deletions and insertions	Leads to a significant reduction in steroidal glycoalkaloid content with a 46% mutation efficiency.	[49]
**CRISPR/Cas system for the reduced of postharvest factors affecting potato**									
Cv. Atlantic and Desiree	*Agrobacterium-*mediated transformation; pFGC-Cas9-ASVI vector	CRISPR/Cas9 system	35S promoter	transgenic	Vacuolar invertase (*VInv*) and asparagine synthetase 1 (*AS1*)	storing potato tubers at cold temperatures	deletions, insertions and substitutions	Reduced fructose and glucose concentrations after cold storage; less acrylamide.	[50]
Cv. Katahdin	*Agrobacterium-*mediated transformation;	CRISPR/Cas9 system	35S promoter	transgenic	Vacuolar invertase (*VInv*)	storing potato tubers at cold temperatures	deletion	Led to a 54% reduction in *VInv* expression in the mutated lines.	[51]
Clone DRH195	*Agrobacterium-*mediated transformation	CRISPR/Cas9 system	*-*	transgenic	Polyphenol oxidases (*StPPO*)	resistance to tuber bruising	indels (insertions and deletions) and SNP	No significant evidence of off-target effects.	[52]
Cvs. Atlantic and Spunta	Protoplast transfection; pTRANS_100 vector	CRISPR/Cas9 system	AtU6 promoter	DNA-free genome editing	Vacuolar invertase (*VInv*) and Polyphenol oxidases (*StPPO*)	resistance to tuber bruising	deletions, insertions and in-frame mutations	Improved chip quality, reduced browning, and largely preserved tuber traits in both gene edited lines.	[53]
Cv. Desiree	Protoplast transfection; pTRANS_100 vector	CRISPR/Cas9 system	*-*	transgenic	Tubulin-like GTPase (*FtsZ1*)	morphology of starch granules	deletions and insertions	Mutated lines showed reduced *FtsZ1* gene expression with increased starch granule size without nutritional quality change.	[54]
**CRISPR/Cas9 system applied for fundamental research**									
Cv. Desiree	protoplasts’ transgene expression and protoplasts’ regeneration	CRISPR/Cas9 system	UBIQUITIN10 promoters	DNA-free gene-editing	Neomycin phosphotransferase2 (*NPT2*)	improving genome editing efficiencies	Not given	Editing efficiency reached to 95%.	[27]
Cv. Desiree	*Agrobacterium*-mediated transformation; a potato virus X (PVX) vector (SlPDS, St&mPDS1, StPDS2, StPDS3, SmPDS2)	CRISPR/Cas9 system	-	transgenic	Phytoene desaturase gene (PDS): *StPDS*	improving genome editing efficiencies	Not given	Editing efficiency increased from 22.1% to 30.5%.	[55]
Cv. Saturna and Wotan	Protoplasts’ transgene expression	CRISPR/Ca system		DNA-free gene-editing	α-glucan water dikinase 1 gene (*GWD1*) and downy mildew resistant 6 (*DMR6-1*) genes	improving genome editing efficiencies	indels and single nucleotide polymorphisms (SNPs)	Resulted significantly better editing in the *GWD1* mutant lines with regions targeted comprising the 5′ end, while editing efficiency was more balanced between the 5′ and 3′ ends the *GWD1* mutant lines.	[56]
Cvs. Desiree and Rywal	*Agrobacterium*-mediated transformation	CRISPR/Cas9 system	-	transgenic	MicroRNAs (*MIR160a*, *MIR160b*, and *MIR390a*)	To establish fast and efficient protocol for CRISPR/Cas9- mediated modulation of miRNA expression	deletions and insertions	Suggesting high editing efficiency.	[57]
many cultivars	*Agrobacterium-*mediated transformation; p2CT-His-MBPLbu_C2c2_WT plasmid vector	CRISPR/Cas13a system	T7 promoter	transgenic	*Clavibacter sepedonicus* detection	to determine viable bacteria of *Clavibacter sepedonicus* in potato tubers	Not given	Effective, easy, time consuming.	[58]

## Data Availability

No new data were created or analyzed in this study.
